# Comparison of Quantiferon Test with Tuberculin Skin Test for the Detection of Tuberculosis Infection in Children

**DOI:** 10.1007/s10753-012-9466-1

**Published:** 2012-04-26

**Authors:** Hatice Onur, Sami Hatipoğlu, Vefik Arıca, Nevin Hatipoğlu, Seçil Gunher Arıca

**Affiliations:** 1 Department of Pediatric, İstanbul Training and Research Hospital, İstanbul, Turkey; 2 The Chair of Pediatric Clinic, Dr Sadi Konuk Training and Research Hospital, İstanbul, Turkey; 3 Department of Pediatric, Mustafa Kemal University Medical Faculty, Serinyol, 31100 Antakya, Hatay Turkey; 4 Department of Infectious Diseases, Kanuni Sultan Süleyman Training and Research Hospital, İstanbul, Turkey; 5 Department of Family Medicine, Mustafa Kemal University Medical Faculty, Antakya, Hatay Turkey

**Keywords:** tuberculosis, quantiferon, tuberculin skin test, BCG

## Abstract

The efficacy of Quantiferon-TB gold test (QFT-GIT) remains to be documented in pediatric population. Tuberculin skin test (TST) is a conventional test available for the diagnosis of latent tuberculosis infection (LTBI). We aimed to investigate the concordance between QFT-GIT and TST in children with and without tuberculosis infection. Ninety-seven patients, aged 3 months–14 years, admitted to pediatric outpatient clinics of Dr. Sadi Konuk Training Hospital Bakırköy, Turkey between March 2008 and April 2009 were recruited. Demographic features, TST results, history of exposure to active tuberculosis (TB), chest X-ray findings, clinical history, presence of Bacillus Calmette Guerin (BCG) vaccination scar were recorded. Patients were categorized into four groups namely, active TB, LTBI, no TB and healthy. It was found that BCG scar positivity did not influence QFT-GIT results. There was a statistically significant agreement between QFT-GIT and TST results (*κ* = 0.486; *p* < 0.01). In patients ≥5 years of age, TST positivity and QFT positivity had a significant relationship (*p* < 0.01). In all patient groups, sensitivity and specificity was 65.85 % and 82.14 %, respectively. In active TB group, TST and QFT-GIT results demonstrated significant agreement ratio of 40.8 % (*κ* = 0.364; *p* < 0.01). Sensitivity and specificity was 100 % and 30 %, respectively. Utilization of QFT-GIT in the diagnosis of LTBI reduces false-positive results and prevents unnecessary treatment with INH and its adverse effects.

## INTRODUCTION

Number of cases with tuberculosis infection increased at the beginning of 21st century. It is reported that one third of world’s population is infected with *Mycobacterium tuberculosis* and 1.7 million deaths worldwide are due to active tuberculosis. It is documented that over 1 million new cases of tuberculosis is diagnosed in children younger than 15 years of age annually and active disease accounts for 400,000 deaths in this population [1]. In Turkey, annual number of new TB cases reported to World Health Organization (WHO) is approximately 20.000; and the incidence of TB is 30/100,000 [1].

TB is diagnosed by combining patient’s history of exposure to active disease, tuberculin skin test (TST) results, clinical and radiological findings. Microbiologic tests yield positive results in only 30–40 % of cases [2]. TST poses some technical problems such as the potential for false-positive and false negative results and problems in administration and interpretation [2].

Identification of Early Secreted Antigenic Target-6 kD protein (ESAT-6) and Culture Filtrate Protein-10 kD (CFT-10) has been a promising development in the diagnosis of TB. These antigenic proteins are encoded within the region of difference 1 (RD 1) of the *M. tuberculosis* genome. Neither of these proteins exist in Bacillus Calmette Guerin (BCG) and non-tuberculosis mycobacteria making them more specific tools in the diagnosis of *M. tuberculosis* [3]. Quantiferon-TB gold test (QFT-GIT), licensed by FDA in 2004, utilizes ESAT-6, CFT-10 and TB7.7 antigens for stimulating *in vitro* release of γ-IFN from memory T cells [4]. Released γ-IFN level is measured by enzyme-linked immunosorbent assay (ELISA) method. Majority of the research about γ-IFN was conducted in countries of low TB incidence and mainly in adults. However, in countries such as Turkey where TB is endemic, more studies are required to document the efficacy of QFT-GIT in pediatric population.

We aimed to investigate the agreement between QFT-GIT and TST results in children with and without tuberculosis infection.

## MATERIALS AND METHODS

Ninety-seven patients admitted to pediatric outpatient clinics of Dr. Sadi Konuk Training Hospital in Bakırköy, Turkey between March 2008 and April 2009 were enrolled. The age of the patients ranged from 3 months to 14 years. Demographic features, TST results, history of exposure to active tuberculosis (TB), chest X-ray findings, clinical history, presence of BCG vaccination scar were recorded. Patients were categorized into four main groups namely, active TB, latent tuberculosis infection (LTBI), no TB, and healthy.

Group I (*n* = 30): Active TB; patients with signs and symptoms suggestive of TB and diagnostic work-up yielded the diagnosis of TB, finally anti-TB chemotherapy started.
Group II (*n* = 15): LTBI, patients were asymptomatic but TST results were positive.
Group III (*n* = 27): Non-TB, patients with signs and symptoms suggestive of TB however diagnostic work-up excluded the disease.
Group IV (*n* = 25): Healthy children.

Written parental informed consent was obtained for each patient. The study was approved by the hospital ethics committee.

### Exclusion Criteria

Patients with hemodynamically unstable cardiopulmonary disease, history of severe allergic reaction to purified protein derivatives (PPD), history of active TB, immunodeficiency, malnutrition and patients whose parent/guardian refused to consent were excluded.

### TST Administration

Tuberculin skin test was performed on the volar aspect of the forearm by administering 0.1 ml (5 tuberculin units) of PPD solution intradermally. Vertical and horizontal diameter of the induration was measured 48–72 h after the injection by using the ball point pen method.

TST result ≥10 mm was considered positive in patients without a BCG vaccination scar and TST result ≥15 mm was considered positive in patients with BCG vaccination scar. In patients with BCG vaccination scars, TST <15 mm was considered negative [5].

### QFT

The QFT assay was performed as per the manufacturer’s instructions. The assay involved two stages: the first stage involved incubation of whole blood with antigens, and the second stage involved measurement of IFN-g production in harvested plasma by ELISA. Venous blood was directly collected, prior to TST administration, into three 1-ml heparin-containing tubes. One tube contained only heparin as negative control, another also contained mitogen as positive control, and the third tube had overlapping peptides representing the entire sequences of ESAT-6 and CFP-10 and another peptide from a portion of the TB antigen TB7.7 (Rv2654). Within 2–6 h of blood draw, the tubes were incubated at 37°C. After exactly 24 h of incubation, the tubes were centrifuged and plasma was harvested and frozen at −70°C until the ELISA was performed (on average, ELISA was performed within 4–6 weeks of blood collection). The γ-INF response was quantified using ELISA (Cellestis Ltd, Carnegie, Victoria, Australia). γ-INF values (in international units per milliliter) for TB-specific antigens and mitogen were corrected for back ground by subtracting the value obtained for the respective negative control. As recommended by the manufacturer, and based on previous studies, the cut-off value for a positive test was γ-INF ≥ 0.35 IU/ml [6, 7].

#### Statistical Analysis

NCSS 2007 and PASS 2008 Statistical Software (Utah, USA) program was used for statistical analysis. Because there is no gold standard for diagnosis of LTBI, concordance between TST and QFT-GIT was assessed by using proportion agreement and kappa coefficients. Chi-square and Fisher’s exact tests were used for categorical variables. A *p* value of less than 0.05 was considered significant and results were reported with 95 % confidence intervals.

## RESULTS

A total of 97 patients were enrolled. Forty eight (49.5 %) patients were female. Age and gender of the patients did not differ significantly (*p* > 0.05; Table 1).

**Table 1:** Demographic and Clinical Features of Patients

	Active TB	LTBI	Healthy	Non-TB	Total	*χ* ^2^; *p*
*N*	30	15	25	27	97	
Gender, *n* (%)						
Female	15 (50%)	8 (53.3%)	10 (40%)	15 (55.6%)	48 (49.5%)	*χ* ^2^ = 1.39; *p* = 0.708
Male	15 (50%)	7 (46.7%)	15 (60%)	12 (44.4%)	49 (50.5%)	
Age, *n* (%)						
<5	9 (30%)	2 (13.3%)	4 (16%)	8 (29.6%)	23 (23.7%)	*χ* ^2^ = 2.89; *p* = 0.408
≥5	21 (70%)	13 (86.7%)	21 (84%)	19 (70.4%)	74 (76.3%)	
QFT, *n* (%)						
Positive	27 (90%)	10 (66.7%)	0 (0%)	0 (0%)	37 (38.1%)	*χ* ^2^ = 73.08; *p* = 0.001**
Negative	3 (10%)	5 (33.3%)	23 (92%)	23 (85.2%)	54 (55.7%)	
Indeterminate	–	–	2 (8%)	4 (14.8%)	(6.2%)	
TST, *n* (%)						
Positive	20 (66.7%)	12 (80%)	0 (0%)	9 (33.3%)	41 (42.3%)	*χ* ^2^ = 35.25; *p* = 0.001**
Negative	10 (33.3%)	3 (20%)	25 (100%)	18 (66.7%)	56 (57.7%)	
BCG vaccination scar, *n* (%)						
Positive	24 (80%)	14 (93.3%)	23 (92%)	24 (88.9%)	85 (87.6%)	*χ* ^2^ = 2.54; *p* = 0.468
Negative	6 (20%)	1 (6.7%)	2 (8%)	3 (11.1%)	12 (12.4%)	
History of exposure, *n* (%)						
Positive	18 (60%)	9 (60%)	0 (0%)	10 (37%)	37 (38.1%)	*χ* ^2^ = 24.54; *p* = 0.001**
Negative	12 (40%)	6 (40%)	25 (100%)	17 (63%)	60 (61.9%)	
Chest X-ray, *n*(%)						
Positive	25 (83.3%)	0 (0%)	–	16 (59.3%)	41 (56.9%)	*χ* ^2^ = 28.42; *p* = 0.001**
Negative	5 (16.7%)	15 (100%)	–	11 (40.7%)	31 (43.1%)	

*Χ* ^2^ Chi-square test

**p < 0.01

**Fig. 1:**
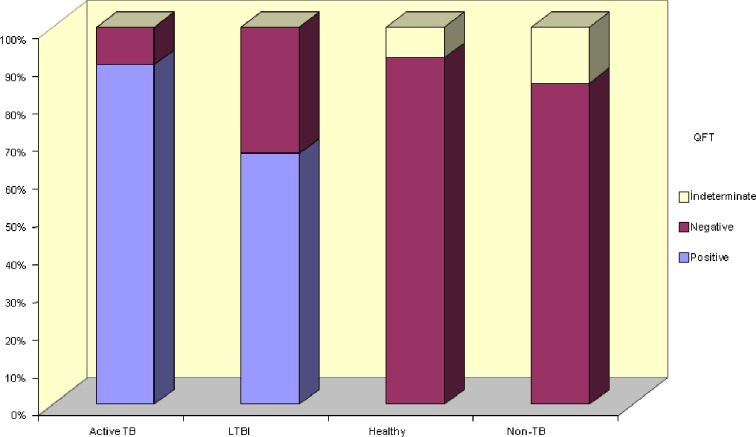
Distribution of QFT results for all groups of patients.

**Fig. 2:**
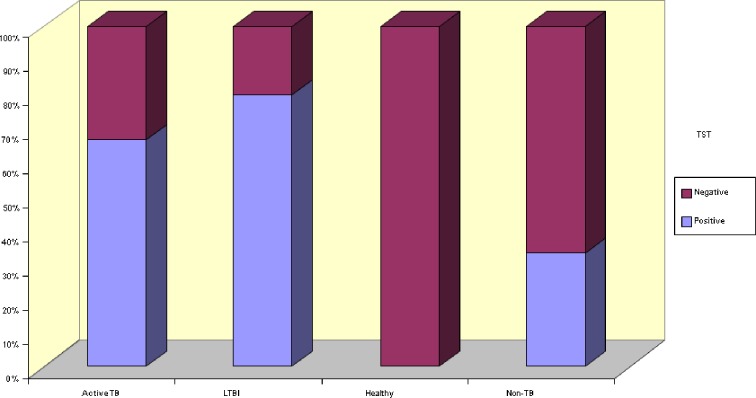
Distribution of TST results for all groups of patients.

Number of positive QFT results differed significantly between groups (*p* < 0.01) QFT positivity rate was significantly higher in group I and II compared to other groups. Rate of indeterminate test results was significantly higher in group III compared to other groups (Fig. 1, Table 1). TST positivity was different between groups (*p* < 0.01). The number of positive TST results was significantly lower in group IV compared to other groups (Fig. 2, Table 1). Numbers of BCG vaccination scar-positive children were close in study groups (*p* > 0.05; Table 1). History of exposure to active TB was significantly different between all groups (*p* < 0.01), it was especially low in group IV (Table 1). Chest X-ray abnormalities were observed in 83.3 %, 0 %, and 59.3 % of cases with active TB, LTBI, and non-TB, respectively (*p* < 0.01; Table 1).

In all cases and groups, presence of BCG vaccination scar influenced neither TST nor QFT results (*p* > 0.05). QFT results were not affected by TST induration diameters; rate of QFT positivity was significantly higher in patients with an induration diameter of 10–14 and ≥15 mm compared to other diameters (Table 2).

**Table 2:** Agreement Between TST and QFT Results

QFT	Diameter of induration (mm)					*p*
	0–5	5–9	10–14	≥15	Total	
	*n* (%)	*n* (%)	*n* (%)	*n* (%)	*n* (%)	
Positive	4 (12.5%)	0 (0%)	7 (50%)	26 (65%)	37 (38.1%)	0.001**
Negative	26 (81.3%)	10 (90.9%)	6 (42.9%)	12 (30%)	54 (55.7%)	
Indeterminate	2 (6.3%)	1 (9.1%)	1 (7.1%)	2 (5%)	6 (6.2%)	
Total	32 (100%)	11 (100%)	14 (100%)	40 (100%)	97 (100%)	

**p < 0.01

In active disease group; QFT results were positive in all patients with positive TST results. In LTBI group, TST positivity was not associated with QFT positivity (*p* > 0.05). In non-TB group, there was not a significant relationship between TST positivity and QFT positivity (*p* > 0.05). When all cases were considered; there was a significant relationship between TST positivity and QFT positivity (*p* < 0.01); TST positivity rate was significantly higher in patients with positive QFT results (Table 3).

**Table 3:** Agreement Between QFT and TST Results

Variable	*n*	Kappa value (*p* value)	Positive agreement %	Negative agreement %	TST/−QFT %	−TCTD/+QFT %	Accuracy (%)
Active TB	29	*κ* = 0.364; *p* = 0.006**	100	30.00	74.07	100	76.67
LTBI	15	*κ* = −0.333; *p* = 0.171	58.33	0	70.0	0	46.67
All cases	97	*κ* = 0.486; *p* = 0.001**	65.85	82.14	72.97	76.67	75.26

**p < 0.01

In active disease group, non-incidental agreement rate between TST and QFT was 40.8 % (*κ* = 0.364; *p* < 0.01). In this group, sensitivity (i.e., rate of positive QFT results in patients with positive TST results) was 100 % and specificity (i.e., rate of negative QFT results in patients with negative TST results) was 30 %. Positive predictive value was 74.07 %; negative predictive value was, accuracy of the test was 76.7 % (Table 3).

In LTBI group, non-incidental agreement rate between TST and QFT was −33.3 % (*κ* = −0.333 *p* > 0.05). In this group sensitivity was 58.33 % and specificity was 0 %. Positive predictive value was 70 %; negative predictive value was 0 %, the accuracy of the test was 46.67 % (Table 3).

In all cases, non-incidental agreement rate between TST and QFT was 48.6 % (*κ* = 0.486; *p* < 0.01). In this group, sensitivity was 65.85 % and specificity was 82.14 %. Positive predictive value was 72.97 %; negative predictive value was 76.67 %, the accuracy of the test was 75.26 % (Table 3).

In patients <5 years of age, there was not a significant relationship between TST positivity and QFT positivity (*p* > 0.05). Non-incidental agreement rate between TST and QFT was 25.1 % (*κ* = 0.251; *p* > 0.05). In this group, sensitivity was 42.85 %; specificity was 92.5 %. Positive predictive value was 50 %; negative predictive value was 90.24 %; the accuracy of the test was 85.1 % (Table 4).

**Table 4:** Agreement of QFT and TST Results According to Age

			TST			*p*
			Positive	Negative	Total	
			*n* (%)	*n* (%)	*n* (%)	
<5 years	QFT	Positive	3 (42.9%)	3 (42.9%)	6 (26.1%)	0.318
		Negative	4 (57.1%)	13 (81.3%)	17 (73.9%)	
		Total	7 (100%)	16 (100%)	30 (100%)	
≥5 years	QFT	Positive	24 (70.6%)	7 (17.5%)	31 (41.9%)	0.001**
		Negative	10 (29.4%)	33 (82.5%)	43 (57.1%)	
		Total	34 (100%)	40 (100%)	74 (100%)	

Fisher’s exact test and Chi-square tests were used

*p < 0.05

In patients ≥5 years of age; there was a significant relationship between TST positivity and QFT positivity (*p* < 0.01); positive TST result rates were significantly higher in patients with positive QFT results. Non-incidental agreement rate between TST and QFT was 53.4 % (*κ* = 0.534; *p* < 0.01). In this group, sensitivity was 70.58 %; specificity was 82.50 %. Positive predictive value was 77.41 %; negative predictive value was 74.74 % and the accuracy of the test was 77.03 % (Table 4)

## DISCUSSION

Recently emerged tests, based on measuring *in vitro* release of IFN-γ from T- lymphocytes upon exposure to mycobacterial antigens, are considered as promising alternative tools to TST in the diagnosis of TB.

Several authors have emphasized that BCG vaccination leads to false positive TST results and they reported that specificity of IFN-γ release assays (IGRA) might be higher [8–10]. TST induration diameter is influenced by the age at which the first BCG dose is received, interval between two doses, and amount of the administered vaccine dose [11, 12]. It is reported that in developed countries, BCG vaccination at birth or during infancy does not affect TST results [7]; however, BCG vaccination affects the sensitivity of TST in underdeveloped and developing countries [1, 13].

It is clearly documented that BCG vaccination does not affect QFT test results [14–18]. Our results parallels the results of previous research and indicates that BCG vaccination status and QFT results do not pose a statistically significant relationship (*p* > 0.05). In populations of widespread BCG vaccine use, QFT may be an alternative tool to TST in the diagnosis of TB.

It is clearly stated in the guideline of Centers for Disease Control and Prevention that since the sensitivities are rather suboptimal, negative QFT and TST results do not rule-out active TB [19]. In various studies comparing TST and QFT, sensitivity was found to be between 55–88 % and mean value of sensitivity was 75 % [20]. Indeterminate results observed in 7 % of patients were attributed to the immunosuppresion due to active TB. It is observed that indeterminant results are detected more frequently in studies encompassing patients with immunodeficiency [20]. In our study, none of the results were indeterminate in patients with active TB. This finding might be attributed to the fact that patients with immunodeficiency were excluded from our study.

In Japan, Kobashi *et al*. conducted a study which included 50 healthy volunteers, 50 patients with active TB, and 100 patients with non-TB infections and emphasized that QFT is superior to TST in differentiating active TB from non-TB *Mycobacterium* infections [21].

Higucki *et al*. compared TST and QFT results of college students who had a history of exposure to an index case with active TB and followed-up these students for 3.5 years. In conclusion, authors recommended utilization of QFT instead of TST while investigating children with a history of exposure to active TB and they proposed that positive TST results should be confirmed via QFT [22].

Mazurek *et al*. published a QFT-G instruction guideline in 2005. In this guide, they reported that QFT-G, similar to TST, was not an effective tool for differentiating LTBI from active TB and stressed the importance of utilizing radiologic examinations, clinical findings, microbiologic, and cytologic investigations for a firmly established diagnosis of TB. The authors reported that neither negative TST results, nor negative QFT results excluded diagnosis of TB infection and added that in the presence of symptoms and signs suggesting diagnosis of TB, negative TST or QFT results might not rule-out TB infection. They also reported the importance of HIV testing in suspicious cases [23]. In our study, non-incidental agreement rate between TST and QFT was −33.3 % (*κ* = −0.333 *p* > 0.05) in LTBI group; sensitivity was 58.33 % and specificity was 0 %. Positive predictive value was 70 %; negative predictive value was 0 %, the accuracy of the test was 46.67 %. TB prevention programs recommend QFT for the follow-up of LTBI in patients with a history of exposure to active disease because QFT makes a second visit unnecessary, is more specific than TST and—contrary to TST—does not have a booster effect [23]. Our findings support this recommendation.

One of the 26 healthy children had a positive QFT result. Although he did not have any signs and symptoms of infection, chest X-ray, and thorax CT findings revealed active TB and anti-tuberculous chemotherapy was commenced promptly. This patient was assigned as healthy when symptoms, history of exposure, TST result and physical examination findings were evaluated. The patient was diagnosed incidentally by QFT. This finding indicates that QFT, as well as TST, may be used in routine screening of TB in our country. Two of the remaining 25 patients had indeterminate results. Since these patients did not have any clinical features suggestive of TB, repeat QFT was not performed as recommended previously [23].

Connel *et al*. stated that rate of QFT positivity and mitogen response increased with advancing age [7]. Nakaoka *et al*. reported a decreased response to QFT in children <5 years of age [24]. Ulrich *et al*. declared that publications indicating a lower response rate to QFT in infants and young children were scarce and more research was needed on that issue [25]. In 2009, Bianchi *et al*. reported that sensitivity of IGRA was higher compared to TST in children less than 4 years of age [26]. In our study, agreement between QFT and TST was 53.4 % (*κ* = 0.251; *p* > 0.05) in patients older than 5 years of age and it was 25.1 % (*κ* = 0.534; *p* < 0.01) in patients younger than 5 years of age. The agreement in patients older than 5 years of age was significant compared to patients younger than 5 years of age. Finally, it can be proposed that QFT may be used routinely for TB monitoring in Turkish children, especially those older than 5 years of age.

It is shown that TST induration diameter and QFT results correlate well [27, 28]. Johnson *et al*. failed to document a significant relationship between TST and QFT results [29]. In our study, QFT positivity differed significantly according to TST induration diameter: QFT positivity rate was significantly higher in patients with an induration diameter of 10–14 and ≥15 mm compared to other diameters (*p* = 0.001). Although QFT-G use is not widespread in our country, children with a TST result between 10 and 14 mm should be followed-up closely.

In literature, a substantially significant agreement was documented between QFT and TST results [10, 30, 31]. We observed in our study that non-incidental agreement between QFT and TST was 48.6 % and it was statistically significant (*κ* = 0.486; *p* < 0.01). In this group, sensitivity was 65.85 % and specificity was 82.14 %. Positive predictive value was 72.97 %; negative predictive value was 76.67 %, the accuracy of the test was 75.26 %.

To conclude, IFN-γ release assays may have numerous advantages over TST such as higher specificity, better relationship with *M. tuberculosis* exposure, and lower cross-reaction rates with BCG vaccination and NTM. Tests based on measurement of released IFN-γ may reduce false-positive results and prevent unnecessary treatment with INH and its adverse effects.
